# *Phyllanthus wightianus* Müll. Arg.: A Potential Source for Natural Antimicrobial Agents

**DOI:** 10.1155/2014/135082

**Published:** 2014-05-05

**Authors:** D. Natarajan, R. Srinivasan, M. S. Shivakumar

**Affiliations:** ^1^Natural Drug Research Laboratory, Department of Biotechnology, Periyar University, Periyar Palkalai Nagar, Salem, Tamil Nadu 636 011, India; ^2^Molecular Entomology Laboratory, Department of Biotechnology, Periyar University, Periyar Palkalai Nagar, Salem, Tamil Nadu 636 011, India

## Abstract

*Phyllanthus wightianus* belongs to Euphorbiaceae family having ethnobotanical importance. The present study deals with validating the antimicrobial potential of solvent leaf extracts of *P. wightianus*. 11 human bacterial pathogens (*Bacillus subtilis*, *Streptococcus pneumoniae*, *Staphylococcus epidermidis*, *Proteus vulgaris*, *Pseudomonas aeruginosa*, *Klebsiella pneumoniae*, *Salmonella typhimurium, Escherichia coli, Shigella flexneri, Proteus vulgaris*, and *Serratia marcescens*) and 4 fungal pathogens (*Candida albicans, Cryptococcus neoformans, Mucor racemosus*, and *Aspergillus niger*) were also challenged with solvent leaf extracts usingagar well and disc diffusion methods. Further, identification of the active component present in the bioactive extract was done using GC-MS analysis. Results show that all extracts exhibited broad spectrum (6–29 mm) of antibacterial activity on most of the tested organisms. The results highlight the fact that the well in agar method was more effective than disc diffusion method. Significant antimicrobial activity was detected in methanol extract against *S. pneumoniae* (29 mm) with MIC and MBC values of 15.62 **μ**g/mL. GC-MS analysis revealed that 29 bioactive constituents were present in methanolic extract of *P. wightianus,* of which 9,12-octadecaenioic acid (peak area 22.82%; RT-23.97) and N-hexadecanoic acid (peak area 21.55% RT-21.796) are the major compounds. The findings of this study show that *P. wightianus* extracts may be used as an anti-infective agent in folklore medicine.

## 1. Introduction

Plants play an important role in human life, primarily, as a source of food and medicine. Man is continuously faced with several lethal infectious diseases caused by pathogenic microorganisms [1]. In recent years, several pathogenic microorganisms have gained resistance to currently available synthetic antimicrobial agents and also caused many health problems [2]. Hence, there is an urgent need to discover an alternative new, broad spectrum, more active, and safer antimicrobial agent. Plant materials remain an important resource to combat serious diseases in the world. Especially, the plants from Pinaceae, Cupressaceae, Apiaceae, Burseraceae, Anacardiaceae, Palmaceae, Euphorbiaceae, Dracenaceae, and Fabaceae families are rich source for antimicrobial agents [3, 4]. Plants have an exceptional ability to synthesize* de novo* antimicrobial agents, in response to microbial attack for its protection [5]. Plant derived natural compounds (such as flavonoids, terpenoids, and steroids) have received considerable attention due to their diverse pharmacological properties including antibacterial and antifungal activities [6].

Antimicrobial components from plants which are mainly secondary metabolites act as inhibitors of bacterial growth, bacterial adherence, exopolysaccharide synthesis, DNA gyrase, cytoplasmic membrane function, and energy metabolism [7, 8]. Berberine, an isoquinoline alkaloid, which is present in roots and stem-bark of* Berberis* species, shows antimicrobial potential against bacteria, fungi, protozoa, and viruses [9]. Diterpene alkaloids, commonly isolated from the plants of the Ranunculaceae family, have antimicrobial properties [10]. Several phenolic compounds such as, caffeic acid, catechol, and pyrogallol are effective antimicrobial agents. The antibacterial activity of some monoterpenes, diterpenoids, sesquiterpenes, triterpenoids, and their derivatives isolated from plants was recently reported [11]. Nowadays, search for plants with antimicrobial activity has evolved [12]. Importance of plants in drug discovery is growing due to vast diversity of the secondary metabolites which possess varied biological activities and act as main source of molecule leading the discovery of new, effective, and safer drugs [13]. Recent attention has been paid to extraction and isolation of biologically active compounds from plant species which are used in herbal medicines [14]. Pharmacognostic investigations of plants or plant extracts were needed to ascertain their biological activities which lead to the discovery of novel drugs or templates for the development of new therapeutic agents [15].

*Phyllanthus wightianus* (Euphorbiaceae) is a monoecious glabrous shrub which grows up to 4.5 m high and is found in the hills (750 to 1000 m) of peninsular India, on the floor and border of shoals, low altitudes in sandveld, hot dry deciduous, mopane woodlands, along banks of seasonal streams, and rivers [16]. It exhibits various biological properties, such as antimicrobial [17–19], larvicidal [20, 21], analgesic [22], wound healing [23], and antioxidant properties [24]. On the basis of the above information, the present investigation was focused on antimicrobial properties of different solvent leaf extracts and GC-MS analysis of bioactive extract of* P. wightianus* Müll. Arg.

## 2. Materials and Methods

### 2.1. Plant Material

Fresh, matured, uninfected leaves of* P. wightianus* were collected from higher altitudes (900–1100 m) of Kolli Hills (latitude 10° 12′–11° 7′ N, longitude 76°–77° 17′ E), Namakkal district, Tamil Nadu, India. The plant material was authenticated by Botanical Survey of India (BSI) (reference number: BSI/SRC/5/23/2013-14/Tech/2081) Coimbatore, Tamil Nadu, India. The voucher specimen (specimen number: PU/BT/NDRL/2010/05) has been deposited in the Natural Drug Research Laboratory, Department of Biotechnology, Periyar University, Salem, Tamil Nadu, India.

### 2.2. Preparation of Extracts

Collected leaves were washed and shade-dried for three weeks and then powdered. The powdered plant material (500 g) was extracted in increasing polarity order (successively) with hexane, chloroform, acetone, ethyl acetate, and methanol in a Soxhlet apparatus for 72 hours. The extractives were filtered through Whatman number 1 filter paper and evaporated under vacuum at 40°C to yield crude extracts.

### 2.3. Used Microorganisms

Three gram positive bacteria, namely,* B. subtilis* (MTCC 441),* S. pneumoniae* (MTCC 655),* S. epidermidis* (MTCC 435), and eight gram negative bacterial strains, such as* P. vulgaris* (MTCC 426),* P. aeruginosa* (MTCC 741),* K. pneumoniae* (MTCC 109),* S. typhimurium* (MTCC 98),* E. coli* (MTCC 739), and* S. flexneri* (MTCC 1457), with two clinical isolates (*P. vulgaris* and* S. marcescens*) and 4 fungal pathogens (*C. albicans, C. neoformans, M. racemosus,* and* A. niger*) were used in this investigation. The fungal strains were obtained from clinical laboratories of Salem District, Tamil Nadu. Each test organism was prepared by inoculating a loop-full of mother culture in a 5 mL of broth (Muller-Hinton broth for bacteria and Sabourd Dextrose broth for fungal cultures) and incubated at appropriate temperature and time for bacterial pathogens (37°C for 16 hours) and fungal strains (room temperature (28°C) for 72 hours). The culture turbidity was adjusted to 0.5 McFarland equivalence (1.5 × 10^8^ CUF) prior to use.

### 2.4. Agar Well Diffusion Method

The agar well diffusion method was employed to determine antibacterial activity of extracts as per the modified method of Natarajan et al. [19]. The standardized test cultures (50 *μ*L) were swabbed on the per-molten Müeller Hinton Agar (MHA) for bacteria and Sabourd Dextrose Agar (SDA) for fungus using aseptic cotton swab. Six wells were made in the seeded plates using sterile cork borer (5 mm diameter). Then, each extract (50 *μ*L = 50 *μ*g) was separately introduced into wells and allowed to diffuse at room temperature. Equal volume of DMSO was served as negative control. About 25 *μ*L of standard antibiotics like fluconazole (fungus) and ciprofloxacin (bacteria) was used as positive control (each 1 *μ*g/*μ*L). The bacterial and fungal plates were incubated at 37°C for 24 hours and at room temperature for 72 hours, respectively. After the incubation period, the zone of growth inhibition was measured (in mm).

### 2.5. Disc Diffusion Method

The disc diffusion test was performed by the method of NCCLS [25] with minor modifications. Test microbial suspension culture (50 *μ*L) was spread on the MHA for bacteria and SDA for fungus by a sterile cotton swab. Sterile discs (5 mm diameter) were loaded with each extract (50 *μ*L) and allowed to dry at room temperature. The dried discs were placed aseptically on the seeded plates. Standard antibiotic discs were used as positive control (gentamicin, vancomycin, and ampicillin for bacteria and fluconazole for fungus (10 mcg/disc)). The plates were incubated as the conditions mentioned in the well diffusion methods and the diameter (in mm) of clear zone of growth inhibition was recorded.

### 2.6. Minimum Inhibitory Concentration (MIC)

The MIC was determined by broth microdilution bioassay method using the modified method of Eloff et al. [26]. MIC was carried out on the basis of antimicrobial results, the extracts which exhibited considerable antimicrobial activity against tested organisms. 100 *μ*L of different concentrations (1–1000 *μ*g/mL) of extracts was introduced into 96-well microplates containing 200 *μ*L of Muller-Hinton broth and 20 *μ*L bacterial cultures were added to each well. The microplate was closed with lid and incubated for 24 h at 37°C. After incubation period, 40 *μ*L of p-iodonitrotetrazolium violet (INT) (0.2 mg/mL) was added to the wells to serve as an indicator of bacterial growth and incubated at 37°C for 1 hour. The minimum inhibitory concentration (MIC) was taken as the lowest concentration of the extract that completely inhibited bacterial growth.

### 2.7. Minimum Bactericidal Concentration (MBC)

The MBC was determined as per method of Khan et al. [27]. MIC test dilutions (5 *μ*l) which showed no color change was subcultured on freshly prepared Mueller Hinton Agar plates and incubated at 37°C for 24 h. The lowest concentration in which no bacterial growth occurred was taken as the Minimum Bactericidal Concentration.

### 2.8. GC-MS Analysis

GC-MS analysis of bioactive extract was carried out on GC Clarus 500 Perkin Elmer system comprising AOC-20i auto sampler. The spectrums of unknown components present in the bioactive extract were identified by compared with known components spectrum which stored in the NIST and WILEY libraries. The name, molecular weight and structure of the components present in the bioactive extract were ascertained. The GC-MS analysis was carried out at Sophisticated Analytical Instrument Facility (SAIF), Indian Institutes of Technology, Chennai, India.

### 2.9. Statistical Analysis

All determinations were done at least in triplicate and averaged. Values were expressed as mean ± standard deviations. Statistical analyses were conducted using SPPS software (16.0 Version). Analysis of variance (ANOVA) in a completely randomized design and Tukey's multiple range tests were used to compare any significant differences between samples. The confident limits used in this study were based on 95% (*P* < 0.05).

## 3. Results and Discussion

The color of extracts and extractive yield of the plant material are presented in Table 1. The results of antimicrobial activity of* P. wightianus* were given in Table 2. All the extracts of* P. wightianus* show broad spectrum antibacterial activity in the range between 6 and 29 mm. The results of agar well diffusion method show that methanolic extract has significant activity against* S. pneumoniae* (29 mm) followed by* S. epidermidis* (17 mm). The considerable amount of antibacterial activity was observed in acetone extract against* S. pneumoniae* (28 mm) followed by* S. flexneri* (12 mm). In disc diffusion method, methanol extract exhibits good antimicrobial activity and the maximum growth inhibition was observed in* S. pneumoniae* (18 mm) followed by* S. epidermidis* (17 mm). Acetone extract having well to moderate antimicrobial activity and maximum activity was detected in* S. epidermidis* (18 mm) followed by* S. pneumoniae* (14 mm). Most of the tested clinical fungal pathogenic strains exhibit no sensitivity on the tested extracts in both agar well and disc diffusion methods. However, high antifungal activity was recorded in methanol extract against* C. neoformans* (10 mm) followed by hexane extract against* M. racemosus* (10 mm) in well diffusion method (Table 2). The overall results highlight that methanol extract exhibits significant activity against most of the tested pathogens compared with other extracts.

The MIC and MBC results (Tables 3 and 4) indicate that the crude extracts of* P. wightianus* inhibited the growth of selected bacterial species. The lowest MIC and MBC value were detected in methanolic extract against* S. pneumoniae* (15.62 *μ*g/mL) and* S. epidermidis* (31.25 *μ*g/mL). The rest of extracts showed moderate to high MIC and MBC values (500–>1000a *μ*g/mL) against most of the tested bacterial pathogens. Plant extracts are considered as having a good inhibitory activity, if they present MICs ≤ 100 *μ*g/mL, a moderate inhibitory activity, if they present MICs ranging from 100 to 500 *μ*g/mL, a weak inhibitory activity, if they present MICs ranging from 500 to 1000 *μ*g/mL, and no inhibitory activity, if they present MICs > 1000a *μ*g/mL [28, 29]. While considering these reports, the MIC and MBC values recorded from antibacterial activity of present investigation might be good to moderate.

The results highlights that significant antibacterial activity was observed in methanol extract against* S. flexneri, S. pneumoniae,* and* S. epidermidis*. Similar observations were made with* P. wightianus* methanolic extract [17]. Our previous findings, report that acetone extract expresses significant activity against most of the clinical bacterial pathogens compared to methanol extract [19]. Whereas, the present results show MTCC bacterial strains to be more sensitive to the methanol extract than acetone extract of* P. wightianus*. This difference in observation may be due to the high concentration (5 mg/well/disc) of extracts being used in the earlier study, whereas, in the present findings, low concentrations of extracts did not inhibit bacterial growth.

Studies have shown that methanolic extracts of many* Phyllanthus* species, such as* P. acidus* [30],* P. muellerianus* [31],* P. amarus, P. maderaspatensis* [32],* P. debilis* [33],* P. amarus, P. emblica* [34], and* P. niruri* [35], harbor promising antimicrobial activity which strengthens the present findings. Several reports stated that methanol is potent solvent for extracting variety of important phytoconstituents, like alkaloids, phenols, tannins, fatty acids, and flavonoids, which harbor antimicrobial potential which support the findings of present investigation [36, 37].

**Figure 1:**
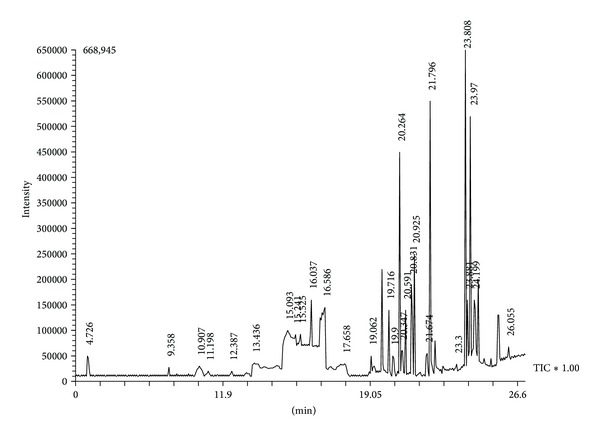
GC-MS chromatogram of methanol leaf extract of* P. wightianus.*

GC-MS analysis shows the presence of 29 compounds which were identified based on their retention time (RT), molecular formula, molecular weight (MW), and concentration (%) (Figure 1 and Table 5). Methnolic extract of* P. wightianus* has 9,12-octadecadienoic acid (with the peak area 22.82% and retention time 23.970) and N-hexadecanoic acid (with the peak area 21.55% and retention time 21.796) acting as major components. A variety of compounds, such as aliphatic ether, aliphatic carboxylic acid, aliphatic ester, alkene, and phenolic compounds were identified. Some of the compounds were present only in low quantities (ranging from 0.6 to 4%).

N-Hexadecanoic acid and 9,12-octadecadienoic acid are common secondary metabolites present in several plants [38–43] and are reported as having many biological properties, like antimicrobial, anti-inflammatory, hypocholesterolemic, cancer preventive, hepatoprotective, and antioxidant [44, 45]. 9,12-Octadecadienoic acid was also stated as a model compound of unsaturated fatty acids, which selectively inhibits FabI enzyme in* S. aureus* and* E. coli*, catalyzing the final and rate-limiting step of the chain elongation process of the type II fatty acid synthesis (FAS-II) in bacteria [46].

Several fatty acids and phenolic compounds were identified in GC-MS analysis of methanol extract which may be the responsible for the antimicrobial activity. The mechanisms of antimicrobial action of fatty acids are nonspecific modes of action [47]. However, antimicrobial effects of fatty acids were observed to form mostly either by a complete inhibition of oxygen uptake or stimulating uptake of amino acids into the cells, which occurs in a dose dependent manner [48]. Fatty acids intercalate in the phospholipid bilayer of microbes due to their lipophilicity, which increases the permeability of the cell membrane, dissipation of the proton-motive force, and leakage of inorganic ions, leading to cell death [49, 50].

Studies have shown that phenolic compounds have bactericidal action by interfering with bacterial cell adhesins, enzymes, cell envelope, and transport proteins [51]. They also increase the free radical concentration within the bacterial protoplasm and irreversibly complex with nucleophilic amino acids in microbial proteins determining loss of their function [52]. As a result, this causes bacterial cell lysis [53]. The antibacterial activity of methanol extract is not only caused by their major compounds, but it could be due to a synergism among their other components present in it. Hence, the presence of these components in higher quantity in methanol extract of* P. wightianus* may be responsible for better bioactivity.

## 4. Conclusions

The findings of present investigation show that agar well diffusion method is ideal for determining the antimicrobial activity of* P. wightianus* extracts. Methanolic extract of* P. wightianus* contributed significant activity against most of the tested bacterial pathogens with least MIC and MBC values. Hence, methanol is the best solvent system for extracting the bioactive principles from leaves of* P. wightianus* which possesses promising antimicrobial principles which may be used in the treatment of infectious diseases caused by pathogenic microbes. The antimicrobial principles from the bioactive extracts may need further purifications to have its synthetic analogues which will be carried out in the future.

## Figures and Tables

**Table 1:** Color and extractive yield of extracts from different solvents of *P.  wightianus*.

Name of extract	Color	Yield (%)
Hexane	Yellowish green	6.24
Chloroform	Dark green	0.67
Acetone	Pale green	1.33
Ethyl acetate	Pale brown	2.49
Methanol	Brown	4.61

**Table 2:** Antimicrobial activity of different solvent extracts of *P. wightianus*.

S. number	Organisms	Method	Diameter of zone of inhibition (in mm)^#^					
			Methanol	Acetone	Ethyl acetate	Chloroform	Hexane	Control*
1	*Bacillus subtilis* (MTCC 441)	Well	09.42 ± 0.52^b^	07.96 ± 0.08^b^	6.66 ± 0.58^b^	00.00 ± 0.00^a^	00.00 ± 0.00^a^	29.74 ± 0.44^c^
		Disc	08.71 ± 0.51^c^	06.53 ± 0.50^b^	00.00 ± 0.00^a^	8.33 ± 0.58^c^	08.33 ± 0.58^c^	15.94 ± 0.10^d^

2	*Escherichia coli* (MTCC 739)	Well	00.00 ± 0.00^a^	00.00 ± 0.00^a^	00.00 ± 0.00^a^	00.00 ± 0.00^a^	00.00 ± 0.00^a^	27.56 ± 0.51^b^
		Disc	07.71 ± 0.51^b^	07.67 ± 0.58^b^	07.62 ± 0.65^b^	08.67 ± 0.58^b^	08.70 ± 0.61^b^	00.00 ± 0.00^a^

3	*Klebsiella pneumoniae* (MTCC 109)	Well	09.52 ± 0.50^b^	10.72 ± 0.63^c^	00.00 ± 0.00^a^	00.00 ± 0.00^a^	00.00 ± 0.00^a^	29.54 ± 0.51^d^
		Disc	09.60 ± 0.36^c^	08.00 ± 0.08^b^	09.83 ± 0.48^c^	08.67 ± 0.58^b,c^	08.72 ± 0.63^b,c^	00.00 ± 0.00^a^

4	*Proteus vulgaris* (MTCC 426)	Well	09.56 ± 0.45^b^	10.96 ± 0.08^c^	00.00 ± 0.00^a^	00.00 ± 0.00^a^	00.00 ± 0.00^a^	24.65 ± 0.36^d^
		Disc	09.82 ± 0.32^d^	08.74 ± 0.65^c,d^	07.67 ± 0.58^b,c^	07.05 ± 0.93^b^	07.51 ± 0.50^b,c^	00.00 ± 0.00^a^

5	*Pseudomonas aeruginosa* (MTCC 741)	Well	12.75 ± 0.66^b^	11.65 ± 0.56^b^	00.00 ± 0.00^a^	00.00 ± 0.00^a^	00.00 ± 0.00^a^	22.67 ± 0.58^c^
		Disc	11.75 ± 0.44^d^	09.83 ± 0.30^c^	6.88 ± 0.43^b^	00.00 ± 0.00^a^	09.64 ± 0.40^c^	00.00 ± 0.00^a^

6	*Salmonella typhimurium* (MTCC 98)	Well	09.59 ± 0.66^c^	11.77 ± 0.68^d^	08.47 ± 0.50^b^	00.00 ± 0.00^a^	08.47 ± 0.50^b^	29.96 ± 0.08^e^
		Disc	09.67 ± 0.58^c^	08.70 ± 0.61^b,c^	08.59 ± 0.71^b,c^	07.27 ± 1.10^b^	07.87 ± 0.23^b^	00.00 ± 0.00^a^

7	*Shigella flexneri* (MTCC 1457)	Well	13.80 ± 0.35^d^	12.77 ± 0.68^d^	07.81 ± 0.33^c^	06.51 ± 0.50^b^	00.00 ± 0.00^a^	15.67 ± 0.58^e^
		Disc	13.84 ± 0.28^d^	11.77 ± 0.68^c^	07.82 ± 0.45^b^	07.78 ± 0.47^b^	08.85 ± 0.25^b^	00.00 ± 0.00^a^

8	*Streptococcus pneumoniae* (MTCC 655)	Well	29.71 ± 0.51^c^	28.55 ± 0.51^c^	10.73 ± 0.64^b^	08.67 ± 0.58^a^	08.00 ± 1.00^a^	34.67 ± 0.58^d^
		Disc	18.85 ± 0.25^d^	14.54 ± 0.50^c^	08.13 ± 0.13^a^	08.67 ± 0.58^a,b^	09.50 ± 0.50^b^	19.52 ± 0.50^d^

9	*Staphylococcus epidermidis* (MTCC 435)	Well	17.83 ± 0.29^b^	10.73 ± 0.64^a^	17.00 ± 1.00^b^	10.67 ± 0.58^a^	11.84 ± 0.77^a^	29.83 ± 0.29^c^
		Disc	17.93 ± 0.12^b^	18.33 ± 0.58^b^	07.67 ± 0.58^a^	07.87 ± 0.23^a^	08.67 ± 0.58^a^	17.80 ± 0.72^b^

10	*Proteus vulgaris *	Well	00.00 ± 0.00^a^	07.77 ± 0.39^b^	00.00 ± 0.00^a^	00.00 ± 0.00^a^	00.00 ± 0.00^a^	21.84 ± 0.27^c^
		Disc	00.00 ± 0.00^a^	00.00 ± 0.00^a^	00.00 ± 0.00^a^	00.00 ± 0.00^a^	00.00 ± 0.00^a^	10.75 ± 0.67^b^

11	*Serratia marcescens *	Well	00.00 ± 0.00^a^	00.00 ± 0.00^a^	08.33 ± 0.32^b^	00.00 ± 0.00^a^	00.00 ± 0.00^a^	24.67 ± 0.58^c^
		Disc	00.00 ± 0.00^a^	00.00 ± 0.00^a^	00.00 ± 0.00^a^	00.00 ± 0.00^a^	00.00 ± 0.00^a^	12.42 ± 1.01^b^

Fungal cultures								
1	*C. albicans *	Well	00.00 ± 0.00^a^	00.00 ± 0.00^a^	00.00 ± 0.00^a^	00.00 ± 0.00^a^	00.000 ± 0.0	00.00 ± 0.00^a^
		Disc	00.00 ± 0.00^a^	00.00 ± 0.00^a^	00.00 ± 0.00^a^	00.00 ± 0.00^a^	00.00 ± 0.00^a^	00.00 ± 0.00^a^

2	*C. neoformans *	Well	10.41 ± 0.52^d^	07.56 ± 0.39^c^	09.78 ± 0.39^d^	06.51 ± 0.50^b^	00.00 ± 0.00^a^	00.00 ± 0.00^a^
		Disc	00.00 ± 0.00^a^	00.00 ± 0.00^a^	00.00 ± 0.00^a^	00.00 ± 0.00^a^	00.00 ± 0.00^a^	27.29 ± 10.3^b^

3	*M. racemosus *	Well	07.67 ± 0.58^b^	09.07 ± 0.40^b,c^	09.67 ± 0.58^b,c^	09.73 ± 0.46^b,c^	10.67 ± 0.58^c^	00.00 ± 0.00^a^
		Disc	00.00 ± 0.00^a^	00.00 ± 0.00^a^	07.93 ± 0.12^c^	06.80 ± 0.35^b^	09.07 ± 0.12^c^	00.00 ± 0.00^a^

4	*A. niger *	Well	09.67 ± 0.58^d^	07.93 ± 0.12^b,c^	09.77 ± 0.40^c,d^	07.77 ± 0.25^b^	09.57 ± 0.51^d^	00.00 ± 0.00^a^
		Disc	07.80 ± 0.35^b,c^	06.67 ± 0.58^b^	07.67 ± 0.58^b,c^	00.00 ± 0.00^a^	08.67 ± 0.58^d^	00.00 ± 0.00^a^

*Standard antibiotics (in well diffusion: ciprofloxacin (1 mg mL^−1^) for bacteria and fluconazole (1 mg mL^−1^) for fungus; in disc diffusion: gentamicin, vancomycin, and ampicillin (10 mcg/disc) for bacteria and fluconazole (10 mcg/disc) for fungus). ^#^The values are mean of triplicates with standard deviations (mean ± S.D; *n* = 3). Different superscript letters (a–e) in rows indicate significant differences (at *P* < 0.05) when subject to Tukey's multiple comparison test.

**Table 3:** MIC (*μ*g/mL) for antibacterial activity of *P. wightianus* against some pathogens.

Organisms	Methanol	Acetone	Ethyl acetate	Chloroform	Hexane	Ciprofloxacin
*Bacillus subtilis* (MTCC 441)	>1000^a^	>1000^a^	>1000^a^	>1000^a^	>1000^a^	3.9^b^

*Escherichia coli* (MTCC 739)	>1000^a^	>1000^a^	>1000^a^	>1000^a^	>1000^a^	15.62^b^

*Klebsiella pneumoniae* (MTCC 109)	500^b^	>1000^a^	>1000^a^	>1000^a^	>1000^a^	7.81^c^

*Proteus vulgaris* (MTCC 426)	>1000^a^	>1000^a^	>1000^a^	>1000^a^	>1000^a^	15.62^b^

*Pseudomonas aeruginosa* (MTCC 741)	250^c^	250^c^	500^b^	>1000^a^	>1000^a^	31.25^d^

*Salmonella typhimurium* (MTCC 98)	500	>1000^a^	>1000^a^	>1000^a^	>1000^a^	15.62

*Shigella flexneri* (MTCC 1457)	125^d^	250^c^	500^b^	>1000^a^	>1000^a^	31.25^e^

*Streptococcus pneumoniae* (MTCC 655)	15.62^d^	31.25^c^	250^b^	>1000^a^	>1000^a^	3.9^e^

*Staphylococcus epidermidis* (MTCC 435)	31.25^d^	62.5^c^	500^b^	>1000^a^	>1000^a^	3.9^c^

The values are mean of triplicates. Different superscript letters (a–e) in rows indicate significant differences (at *P* < 0.05) when subject to Tukey's multiple comparison test.

**Table 4:** MBC (*μ*g/mL) for antibacterial activity of *P. wightianus* against some pathogens.

Organisms	Methanol	Acetone	Ethyl acetate	Chloroform	Hexane	Ciprofloxacin
*Bacillus subtilis* (MTCC 441)	>1000^a^	>1000^a^	>1000^a^	>1000^a^	>1000^a^	15.62^b^

*Escherichia coli* (MTCC 739)	>1000^a^	>1000^a^	>1000^a^	>1000^a^	>1000^a^	31.25^b^

*Klebsiella pneumoniae* (MTCC 109)	>1000^a^	>1000^a^	>1000^a^	>1000^a^	>1000^a^	15.62^b^

*Proteus vulgaris* (MTCC 426)	>1000^a^	>1000^a^	>1000^a^	>1000^a^	>1000^a^	31.25^b^

*Pseudomonas aeruginosa* (MTCC 741)	500^b^	500^b^	500^b^	>1000^a^	>1000^a^	31.25^c^

*Salmonella typhimurium* (MTCC 98)	500^b^	>1000^a^	>1000^a^	>1000^a^	>1000^a^	31.25^c^

*Shigella flexneri* (MTCC 1457)	250^c^	250^c^	500^b^	>1000^a^	>1000^a^	62.5^d^

*Streptococcus pneumoniae* (MTCC 655)	15.62^d^	62.5^c^	500^b^	>1000^a^	>1000^a^	3.9^e^

*Staphylococcus epidermidis* (MTCC 435)	31.25^c^	125^b^	1000^a^	>1000^a^	>1000^a^	3.9^d^

The values are mean of triplicates. Different superscript letters (a–e) in rows indicate significant differences (at *P* < 0.05) when subject to Tukey's multiple comparison test.

**Table 5:** Compounds detected in methanol leaf extract of *P. wightianus* using GC-MS analysis.

Peak	R. time	Peak area (%)	Molecular formula	Molecular weight	Compound name
1	4.726	0.94	C_6_H_12_O_2_	116	2-Pentanone, 4-hydroxy-4-methyl-3 2-hydroxy-2-methyl-4-pentanone 2-

2	9.358	0.28	C_6_H_6_O_3_	126	Levoglucosenone 6,8-ioxabicyclooct-2-en-4-one

3	10.907	0.67	C_8_H_8_O	120	4-Vinylphenol

4	11.198	0.26	C_8_H_18_O_2_	174	T-Butyldimethylsilyl acetate

5	12.387	0.08	C_13_H_16_O_2_	204	1-(4-Methoxyphenyl)-5-hexen-1-one

6	13.436	1.60	C_6_H_6_O_3_	126	1,2,3-Benzenetriol (pyrogallol)

7	15.093	6.07	C_15_H_24_O_2_	236	Butanoic acid, 3-methyl-, 3,7 dimethyl-2,4,6-octatrienyl ester

8	15.241	3.56	C_15_H_20_Br_2_O_2_	390	3,8-Dioxabicyclononane, 6-bromo-4-(1-bromopropyl)-2-[2-penten-4-ynyl]

9	15.525	1.04	C_14_H_22_O	206	Phenol, 2,4-bis(1,1-dimethylethyl)-2,4-ditert-butylphenol 1-hydroxy-2, 4-di-tert-butylbenze

10	16.037	2.46	C_11_H_16_O_2_	180	2-Benzofuranone, 5,6,7,7a-tetrahydro-4,4,7a-trimethyl- (2,6,6-trimethyl-2-hydroxycyclohexylidene)acetic acid lactone 4,5

11	16.586	0.55	C_12_H_14_O_4 _	222	1,2-Benzenedicarboxylic acid, diethyl ester phthalic acid

12	17.658	0.77	C_21_H_32_O_3_	332	*β*-Dodecahydro

13	19.062	0.71	C_11_H_16_O_3_	196	2(4h)-Benzofuranone, 5,6,7,7a-tetrahydro-6-hydroxy-4,4,7a-trimethyl-, (6s-cis)-(—)-loliolide

14	19.716	3.03	C_13_H_20_O_2_	208	Pluchidiol

15	20.264	10.65	C_20_H_38_	278	2,6,10-Trimethyl,14-ethylene-14-pentadecne

16	20.347	1.19	C_18_H_36_O	268	2-Pentadecanone, 6,10,14-trimethyl- hexahydrofarnesyl acetone 6,10,14

17	20.591	3.16	C_20_H_40_	296	3,7,11,15-Tetramethyl-2-hexadecen-1-ol (2e)-3,7,11,15-tetramethyl

18	20.831	5.85	C_20_H_40_	296	3,7,11,15-Tetramethyl-2-hexadecen-1-ol (2e)-3,7,11,15-tetramethyl

19	20.925	0.36	C_24_H_34_O_2_	446	Butanal

20	21.674	0.58	C_10_H_20_	156	6-Octen-1-ol, 3,7-dimethyl-

21	21.796	21.55	C_16_H_32_O_2_	256	N-Hexadecanoic acid, palmitic acid, pentadecanecarboxylic acid

22	23.300	0.53	C_18_H_38_O	270	Bis-(3,5,5-trimethylhexyl) ether

23	23.808	0.40	C_20_H_38_	278	2,6,10-Trimethyl,14-ethylene-14-pentadecne neophytadiene

24	23.881	3.81	C_11_H_22_O_2_	186	Nonanoic acid, 7-methyl ester methyl 7-methylnonanoate

25	23.970	22.82	C_18_H_32_O_2_	280	9,12-Octadecadienoic acid-linoleic acid grape seed oil linoleic linole

26	24.199	5.93	C_18_H_30_O_2_	278	9,12,15-Octadecatrienoic acid, linolenic acids alpha-linolenic acid

27	24.308	0.68	C_18_H_36_O_2_	284	Octadecanoic acid (stearic acid)

28	26.055	0.46	C_8_H_14_O	126	(3z)-6-methyl-3,6-heptadien-1-ol

## References

[1] Das S, Sarkar A, Seth A, Gupta N, Agrawal RC (2012). Evaluation of *in-vitro* antibacterial potential of ripe fruits of *Aegle marmelos*. *International Journal of Pharmacy and Pharmaceutical Sciences*.

[2] Doudach L, Meddah B, Alnamer R, Chibani F, Cherrah Y (2012). *In vitro* antibacterial activity of the methanolic and aqueous extracts of *Anacyclus pyrethrum* used in Moroccan traditional medicine. *International Journal of Pharmacy and Pharmaceutical Sciences*.

[3] Termentzi A, Fokialakis N, Skaltsounis AL (2011). Natural resins and bioactive natural products thereof as potential anitimicrobial agents. *Current Pharmaceutical Design*.

[4] Paraschos S, Mitakou S, Skaltsounis AL (2012). Chios gum mastic: a review of its biological activities. *Current Medicinal Chemistry*.

[5] Gibbons S (2005). Plants as a source of bacterial resistance modulators and anti-infective agents. *Phytochemistry Reviews*.

[6] Singha PK, Roy S, Dey S (2003). Antimicrobial activity of *Andrographis paniculata*. *Fitoterapia*.

[7] Bakkali F, Averbeck S, Averbeck D, Idaomar M (2008). Biological effects of essential oils—a review. *Food and Chemical Toxicology*.

[8] Jeon J-G, Rosalen PL, Falsetta ML, Koo H (2011). Natural products in caries research: current (limited) knowledge, challenges and future perspective. *Caries Research*.

[9] Kim SH, Lee SJ, Lee JH, Sun WS, Kim JH (2002). Antimicrobial activity of 9-*O*-acyl- and 9-*O*-alkylberberrubine derivatives. *Planta Medica*.

[10] Attar-ur-Rahman, Choudhary MI (1995). Diterpenoid and steroidal alkaloids. *Natural Product Reports*.

[11] Kurek A, Grudniak AM, Kraczkiewicz-Dowjat A, Wolska KI (2011). New antibacterial therapeutics and strategies. *Polish Journal of Microbiology*.

[12] Vermani K, Garg S (2002). Herbal medicines for sexually transmitted diseases and AIDS. *Journal of Ethnopharmacology*.

[13] Ahmad I, Mehmood Z, Mohammad F (1998). Screening of some Indian medicinal plants for their antimicrobial properties. *Journal of Ethnopharmacology*.

[14] Essawi T, Srour M (2000). Screening of some Palestinian medicinal plants for antibacterial activity. *Journal of Ethnopharmacology*.

[15] Natarajan D, Shivakumar MS, Srinivasan R (2010). Antibacterial activity of leaf extracts of *Biophytum sensitivum* (L.) DC. *Journal of Pharmaceutical Sciences and Research*.

[16] Radcliffe-Smith A, Pope GV (1999). Euphorbiaceae. *Flora Zambesiaca*.

[17] Mohanasundari C, Natarajan D, Srinivasan K, Anbuganapathi G, Gowrishankar J, Perumal G (2005). Antibacterial efficacy of leaf extracts of *Phyllanthus wightianus* Müll. Arg. *Journal of Phytological Research*.

[18] Sengottuvel R, Srinivasan K, Mohanasundari C, Natarajan D, Perumal G (2007). Screening of antimicrobial properties of leaf extracts of *Smilax zeylanica* and *Phyllanthus wightianus*. *Advances in Plant Sciences*.

[19] Natarajan D, Srinivasan R, Shivakumar MS (2012). *Phyllanthus wightianus* Müll. Arg.: a potential source for antibacterial and phytochemical analysis. *International Journal of Pharmacy and Pharmaceutical Sciences*.

[20] Srinivasan R, Shivakumar MS, Natarajan D, Tyagi BK Larvicidal efficacy of *Phyllanthus wightianus* Müll. Arg. on *Aedes aegypti*.

[21] Shivakumar MS, Srinivasan R, Natarajan D (2013). Larvicidal potential of some Indian medicinal plant extracts against *Aedes aegypti* (L.). *Asian Journal of Pharmaceutical and Clinical Research*.

[22] Valarmathi R, Scnthamarai R, Akilandeswari S (2009). Analgesic and antimicrobial activity of the dried root extracts of *Reidia floribunda* Wight. *Hamdard Medicus*.

[23] Priya OS Wound healing activity of *Phyllanthus wightianus*.

[24] Priya OS, Viswanathan MBG, Balakrishna K, Venkatesan M (2011). Chemical constituents and *in vitro* antioxidant activity of *Phyllanthus wightianus*. *Natural Product Research*.

[25] National Committee for Clinical Laboratory Standards (NCCLS) (2006). *Methods for Dilution Antimicrobial Susceptibility Tests for Bacteria that Grow Aerobically*.

[26] Eloff JN, Katerere DR, McGaw LJ (2008). The biological activity and chemistry of the southern African Combretaceae. *Journal of Ethnopharmacology*.

[27] Khan R, Islam B, Akram M (2009). Antimicrobial activity of five herbal extracts against Multi Drug Resistant (MDR) strains of bacteria and fungus of clinical origin. *Molecules*.

[28] Tanaka JCA, da Silva CC, Filho BPD, Nakamura CV, de Carvalho JE, Foglio MA (2005). Chemical constituents of *Luehea divaricata* Mart. (Tiliaceae). *Quimica Nova*.

[29] Dall’Agnol R, Ferraz A, Bernardi AP (2003). Antimicrobial activity of some *Hypericum* species. *Phytomedicine*.

[30] Jagessar RC, Mars A, Gomes G (2008). Selective Antimicrobial properties of *Phyllanthus acidus* leaf extract against *Candida albicans*, *Escherichia coli* and *Staphylococcus aureus* using Stokes Disc diffusion, Well diffusion, Streak plate and a dilution method. *Natural Science*.

[31] Onocha PA, Opegbemi AO, Kadri AO, Ajayi KM, Okorie DA (2003). Euphorbiaceae plants 1: *Phyllanthus amarus* and *Phyllanthus muellerianus* leaf extracts. *Nigerian Journal of Natural Product of Medicine*.

[32] Ashokkumar R, Ramaswamy M (2013). Comparative study on the antimicrobial activity of leaf extracts of four selected Indian medicinal plants against *Pseudomonas aeruginosa*, *Pseudomonas fluorescens*, *Penicillium chrysogenum* and *Penicillium restrictum*. *Journal of Chemical Biology Physical Science*.

[33] Chandrashekar KS, Satyanarayana, Prasanna KS (2011). Antimicrobial activity of *Phyllanthus debilis*. *International Research Journal of Pharmacy*.

[34] de Britto J, Gracelin DHS, Sebastian SR (2011). Antibacterial activity of a few medicinal plants against *Xanthomonas campestris* and *Aeromonas hydrophila*. *Journal of Biopesticides*.

[35] Mathur M, Sharma R, Sharma J, Pareek R, Kamal R (2012). Phytochemical screening and antimicrobial activity of *Phyllanthus niruri* Linn. *Applied Botany*.

[36] Sivaraj R, Balakrishnan A, Thenmozhi M, Venckatesh R (2011). Preliminary phytochemical analysis of *Aegle marmelos*, *Ruta graveolens*, *Opuntia dellini*, *Euphorbia royleana* and *Euphorbia antiquoru*. *International Journal of Pharmaceutical Science Research*.

[37] Proestos C, Chorianopoulos N, Nychas E, Komaitis M (2005). RP-HPLC analysis of the phenolic compounds of plant extracts. Investigation of their antioxidant capacity and antimicrobial activity. *Journal of Agricultural and Food Chemistry*.

[38] Lee J-C, Kim H-R, Kim J, Jang Y-S (2002). Antioxidant property of an ethanol extract of the stem of *Opuntia ficus-indica* var. *Saboten*. *Journal of Agricultural and Food Chemistry*.

[39] Sermakkani M, Thangapandian V (2012). GC-MS analysis of *Cassia italica* leaf methanol extract. *Asian Journal of Pharmaceutical Clinical Research*.

[40] Al-Shammari AL, Hassan HBW, Al-Youssef AH (2012). Chemical composition and antimicrobial activity of the essential oil and lipid content of *Carduus pycnocephalus* L. growing in Saudi Arabia. *Journal of Chemical and Pharmaceutical Research*.

[41] Albishri HM, Almaghrabi OA, Moussa TAA (2013). Characterization and chemical composition of fatty acids content of watermelon and muskmelon cultivars in Saudi Arabia using gas chromatography/mass spectroscopy. *Pharmacongnosy Magazine*.

[42] Preethi RV, Devanathan V, Loganathan M (2010). Antimicrobial and antioxidant efficacy of some medicinal plants against food borne pathogens. *Advananced Biological Research*.

[43] Rajeswari G, Murugan M, Mohan VR (2013). GC-MS analysis of bioactive components of *Hugonia mystax* L. (Linaceae). *Research Journal of Pharmaceutical, Biological and Chemical Sciences*.

[44] Gunstone FD, Harwood JL, Padley FB (1994). *The Lipid Handbook*.

[45] Maruthupandian A, Mohan VR (2011). GC-MS analysis of some bioactive constituents of *Pterocarpus marsupium* Roxb. *International Journal of Chemical Technology Research*.

[46] Zheng CJ, Yoo J-S, Lee T-G, Cho H-Y, Kim Y-H, Kim W-G (2005). Fatty acid synthesis is a target for antibacterial activity of unsaturated fatty acids. *FEBS Letters*.

[47] Davidson PM, Sofos JN, Branen AL (2005). *Antimicrobials in Foods*.

[48] Orhan I, Özçelik B, Şener B (2011). Evaluation of antibacterial, antifungal, antiviral, and antioxidant potentials of some edible oils and their fatty acid profiles. *Turkish Journal of Biology*.

[49] Souza EL, Oliveira CEV, Stamford TLM, Conceicao ML, Neto NJG (2013). Influence of carvacrol and thymol on the physiological attributes, enterotoxin production and surface characteristics of *Staphylococcus aureus*strains isolated from foods. *Brazilian Journal of Microbiology*.

[50] Lambert RJW, Skandamis PN, Coote PJ, Nychas GJE (2001). A study of the minimum inhibitory concentration and mode of action of oregano essential oil, thymol and carvacrol. *Journal of Applied Microbiology*.

[51] Cazarolli LH, Zanatta L, Alberton EH (2008). Flavonoids: prospective drug candidates. *Mini-Reviews in Medicinal Chemistry*.

[52] Saleem M, Nazir M, Ali MS (2010). Antimicrobial natural products: an update on future antibiotic drug candidates. *Natural Product Reports*.

[53] Engels C, Schieber A, Gänzle MG (2011). Inhibitory spectra and modes of antimicrobial action of gallotannins from mango kernels (*Mangifera indica* L.). *Applied and Environmental Microbiology*.

